# Treatment of an Intractable Forefoot Ulcer Using Realignment Osteotomy in a Patient with Rheumatoid Arthritis

**DOI:** 10.1155/2020/8817456

**Published:** 2020-07-27

**Authors:** Makoto Imai, Naoki Kondo, Rei Kumazaki, Naoto Endo

**Affiliations:** Division of Orthopedic Surgery, Department of Regenerative and Transplant Medicine, Niigata University Graduate School of Medical and Dental Sciences, Japan

## Abstract

Forefoot deformities are common among patients with rheumatoid arthritis (RA). Herein, we describe a case of intractable ulceration on the dorsomedial aspect of the right 5^th^ digit, secondary to forefoot deformity, in a 76-year-old woman with a 35-year history of RA. The ulcer was due to a persistent subcutaneous infection. Although the infection was controllable with antibiotics, there was concern of relapse because of the abnormal pressure on the skin due to an overlap of the 4^th^ and 5^th^ digits. We proceeded with surgical correction of the forefoot alignment, including shortening oblique osteotomy of metatarsals 2 through 5, rather than amputation of the 5^th^ digit. Following surgery, targeted antibiotic treatment was provided. The postoperative course was unremarkable, and the patient recovered weight-bearing function without recurrence of pain or ulceration. Forefoot realignment is a feasible option that should be considered for treating intractable foot pain and ulceration secondary to long-lasting RA.

## 1. Introduction

Forefoot deformities commonly develop in patients with rheumatoid arthritis (RA). It is estimated that two-thirds of patients with chronic polyarthritis will develop subluxation and dislocation of the metatarsal phalangeal (MTP) joints [1]. The resulting overlap between the digits of the foot can lead to ulceration secondary to the abnormal pressure on the skin. Although such ulcers can often be effectively treated conservatively, herein, we present the case of an intractable ulcer of the 5^th^ digit caused by an overlap between the 4^th^ and 5^th^ digits of the foot. We describe our successful treatment of this ulcer by surgical correction of the forefoot alignment.

## 2. Case Presentation

The patient was a 76-year-old woman with a 35-year history of RA, presenting with a long-standing ulcer on the dorsal aspect of her right 5^th^ digit (small toe). Various conservative treatment strategies were used to manage this long-lasting ulcer as follows. At the age of 52 years, she was treated with the direct application of a gold preparation, salazosulfapyridine, to the ulcer. At the age of 57 years, the treatment was changed to prednisolone (7.5 mg/day), with methotrexate (4 mg/week) added at the age of 58 years. At the age of 61 years, the methotrexate dose was increased to 6 mg/week. The prednisolone dose was reduced to 5 mg/day at the age of 73 years and was discontinued at 76 years of age due to the development of pleurisy as a complication.

Comorbidities included a cerebral aneurysm, hypertension, and a fracture of the 12^th^ thoracic vertebra (T12). She had been treated with 200 mg/day of cilostazol as anticoagulant therapy. Following this vertebral fracture, the patient was mostly confined to a wheelchair, although she retained capacity for active movement of her lower limbs and for some weight-bearing through her lower limbs for standing.

**Figure 1:**
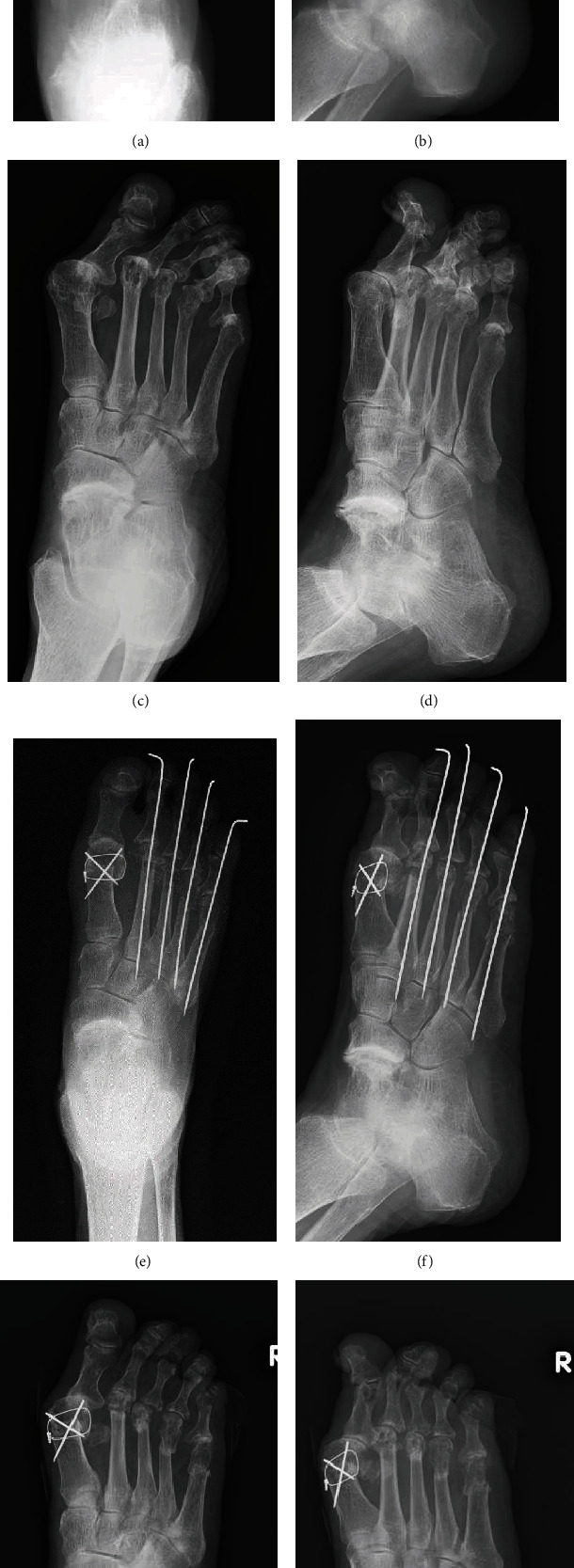
Time course of radiographs of the right foot. At 61 years old, X-ray shows that the hallux valgus angle (HVA) is 29°, the interphalangeal angle between the first and second metatarsals (M_1_M_2_A) is 9°, and the interphalangeal angle between the first and fifth metatarsals (M_1_M_5_A) is 30° (a; anteroposterior view) (b; oblique view). Just before surgery at 76 years old, HVA is 50°, M_1_M_2_A is 15°, and M_1_M_5_A is 35°, respectively (c; anteroposterior view) (d; oblique view). Mitchell's osteotomy for hallux and shortening oblique osteotomies for lesser toes were performed. Postoperative radiographs show that HVA is 19°, M_1_M_2_A is 7°, and M_1_M_5_A is 23°, respectively (e; anteroposterior view) (f; oblique view). Recent follow-up radiographs at 6 months after the surgery show that temporal Kirschner wires for lesser toes are removed and that each osteotomy site healed (g; anteroposterior view) (h; oblique view).

**Figure 2:**
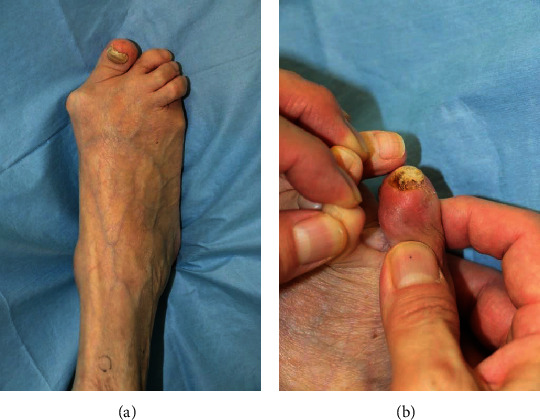
Preoperative macroscopic findings of the dorsum of the right foot (a), with an observable ulcer on the dorsomedial aspect of the 5^th^ digit (b).

Her history of her severe forefoot deformity was as follows. At the age of 61 years, there was no observable dislocation of the MTP joints, with relevant radiographic measures of forefoot alignment as follows: hallux valgus angle (HVA), 29°; angle between metatarsals 1 and 2 (M_1_M_2_A), 9°; and angle between metatarsals 1 and 5 (M_1_M_5_A), 30° (Figures 1(a) and 1(b)). At the age of 76 years, these angles had progressed as follows: HVA, 50°; M_1_M_2_A, 15°; and M_1_M_5_A, 35° (Figures 1(c) and 1(d)). A valgus deformity of the forefoot, including the 2^nd^ and 4^th^ digits, was observed, with a varus deformity of the 5^th^ digit (Figure 2(a)). Her Disease Activity Score (DAS-28) was 3.38 (moderate disease activity), with a Japanese Society for Surgery of the Foot (RA foot ankle scale) score [2] of 53 (per 100 points in maximum).

Persistent redness and swelling of the 5^th^ digit were diagnosed as a subcutaneous infection (Figure 2(b)), and intravenous (IV) piperacillin/tazobactam treatment at a dose of 4.5 g every 8 h was initiated. One week after initiating antibiotic treatment, the redness improved, and the antibiotic was changed to oral minocycline for 2 weeks. Although antibiotic therapy controlled the infection, there was no improvement in the skin ulcer on the dorsum of the 5^th^ digit, and thus, the possibility of recurrent infection remained. The following three treatment options were considered: continued treatment of recurrent infections, wound debridement, and amputation of the 5^th^ digit. As options 1 and 2 are associated with persistent risk of infection due to continued pressure on the skin and option 3 is highly invasive, none of these were selected. Rather, we decided to proceed with surgical correction of forefoot alignment, including correction of hallux valgus, using Mitchell's osteotomy [3], and correction of the overlap between the 4^th^ and 5^th^ digits using a shortening oblique osteotomy of metatarsals 2 through 5 (Figures 1(e) and 1(f)). HVA was improved from 50° to 19°, M_1_M_2_A was improved from 15° to 7°, and M_1_M_5_A was also improved from 35° to 23°.

The surgery was completed in 2 h 39 min, with a blood loss of 75 ml. With the identification of *Staphylococcus* as the causative infectious bacteria, flomoxef, at a dose of 1 g every 8 h, was administered for 2 weeks after surgery to prevent surgical site infection. Subsequently, oral cefditoren pivoxyl therapy (250 mg, once after each meal) was initiated. Sutures were removed at the surgical site 2 weeks after surgery.

**Figure 3:**
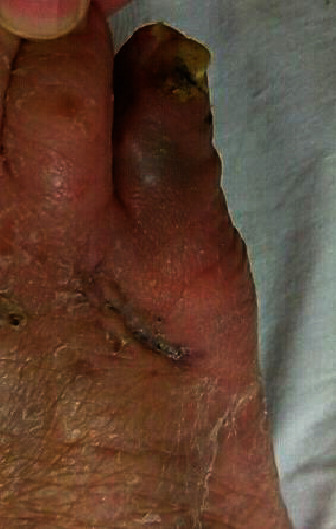
Postoperative macroscopic findings of the right foot, showing complete resolution of the ulcer.

On postoperative day 16, pus discharge was observed from the dorsolateral aspect of the 5^th^ digit of the right foot. Bacterial culture identified *Finegoldia magna*, and accordingly, the antimicrobial therapy was changed to oral minocycline (100 mg, once after breakfast and dinner). The Kirschner wires were removed 3 weeks postsurgery, with no radiographic evidence of osteomyelitis of the proximal and distal phalanges of the 5^th^ digit. On postoperative day 30, redness and discharge from the wound were no longer observable (Figure 3). The patient was able to wear a shoe with an arch support and was able to stand unsupported. Her Japanese Society for Surgery of the Foot (RA foot ankle scale) score increased to 62 points. In particular, her deformity subscale increased from 15 to 25 points. No infectious sign was detected at 6 months after the surgery. Radiograph of right foot demonstrated healing at all the osteotomy site, and overlapping between the 4^th^ and 5^th^ toes was no longer detected at 6 months after the surgery (Figures 1(g) and 1(h)).

## 3. Discussion

In the present case, the benefits acquired from foot surgery (the procedure was Mitchell's osteotomy and shortening oblique osteotomy) were (1) intractable foot ulcer was totally cured, (2) flattened transverse arch improved and metatarsalgia on walking (or weight-bearing) reduced, and (3) cosmesis improved.

Lower extremity ulceration is a common complication of long-standing RA [4], with active RA disease and glucocorticoid therapy being risk factors for foot ulcers [5]. Foot deformity and ill-fitting shoes and appliances can further predispose these patients to ulcers due to local trauma or abnormal skin pressure [6]. Commonly, wound debridement or amputation has been considered for patients presenting with intractable foot ulcers. In our case, we did not proceed with wound debridement owing to the persistent local pressure on the skin due to the overlap between the 4^th^ and 5^th^ digits. Amputation of the 5^th^ digit was considered a possible solution to eliminate the local pressure but was not deemed as an appropriate treatment due to issues of ethics and cosmesis. Alternately, we proceeded with the correction of the hallux valgus deformity and correction of the alignment of the forefoot as a strategy to eliminate the abnormal pressure on the dorsum of the 5^th^ digit, thus allowing for the healing of the ulcer and possible improvement in activities of daily living. Postsurgery, the ulcer healed with no recurrence of infection. Furthermore, foot cosmesis was improved, and the patient could stand independently for transfers from her wheelchair.

Shortening oblique osteotomy (SOO) is a useful procedure to improve the weight-bearing distribution of the foot in patients with RA. Several types of metatarsal shortening osteotomies with MTP joint preservation have been described [7–12]. Hirao et al. reported that the alignment score for the 2^nd^ through 5^th^ digits on the Japanese Society for Surgery of the Foot scale significantly improved by modified metatarsal shortening offset osteotomy from 1.2 ± 2.9 preoperatively to 11.8 ± 3.6 postoperatively in patients with RA foot [7]. Nishida et al. demonstrated that the deformity score for JSSF RA ankle and foot scale significantly improved from 13.8 to 21.1 [8]. In the present case, the deformity score also improved from 15 to 25 points. Delayed wound healing, bony ankylosis of the MTP joint, and recurrent MTP joint subluxation/dislocation of the 5^th^ digit have been reported after SOO [8]. The possibility of postoperative metatarsalgia was considered owing to the poor balance on the other toes. In the present case, the patient had no postoperative metatarsalgia. Moreover, as the hallux valgus deformity was in a sustained state, we were concerned about the risk of increasing pain and callosity formation over time. For these reasons, we proceeded with correction of the alignment of all the digits of the right foot, as well as correction of both the transverse and longitudinal arches. Following correction of the forefoot alignment, there has been no recurrence of foot pain or callosity formation.

Our case indicates that correction of the forefoot alignment in patients with a foot deformity associated with prolonged RA can be useful to improve cosmesis and function. In the case we describe herein, correction of the forefoot alignment further provided permanent treatment for an intractable ulcer of the 5^th^ digit associated with recurrent infection.

## Data Availability

The data that support the findings of this study are available from the corresponding author, NK, upon reasonable request.

## References

[1] Louwerens L. W., Schrier J. C. (2013). Rheumatoid forefoot deformity: pathophysiology, evaluation and operative treatment options. *International Orthopaedics*.

[2] Niki H., Aoki H., Inokuchi S. (2005). Development reliability of a standard rating system for outcome measurement of foot ankle disorders I: development of standard rating system. *Journal of Orthopaedic Science*.

[3] Mitchell C. L., Fleming J. L., Allen R., Glenney C., Sanford G. A. (1958). Osteotomy-bunionectomy for hallux valgus. *The Journal of Bone & Joint SurgeryThe Journal of Bone and Joint Surgery. American Volume*.

[4] Jebakumar A. J., Udayakumar P. D., Crowson C. S., Gabriel S. E., Matteson E. L. (2014). Occurrence and effect of lower extremity ulcer in rheumatoid arthritis — a population-based study. *The Journal of Rheumatology*.

[5] Firth J., Helliwell P., Hale C., Hill J., Nelson E. A. (2008). The predictors of foot ulceration in patients with rheumatoid arthritis: a preliminary investigation. *Clinical Rheumatology*.

[6] Pun Y. L., Barraclough D. R., Muirden K. D. (1990). Leg ulcers in rheumatoid arthritis. *The Medical Journal of Australia*.

[7] Hirao M., Ebina K., Tsuboi H. (2017). Outcomes of modified metatarsal shortening offset osteotomy for forefoot deformity in patients with rheumatoid arthritis: short to mid-term follow-up. *Modern Rheumatology*.

[8] Nishida K., Machida T., Horita M. (2016). Shortening oblique osteotomy with screw fixation for correction of the lesser metatarsophalangeal joints of rheumatoid forefoot. *Acta Medica Okayama*.

[9] Biz C., Corradin M., Kuete Kanah W. T. (2018). Medium-long-term clinical and radiographic outcomes of minimally invasive distal metatarsal metaphyseal osteotomy (DMMO) for central primary metatarsalgia: Do Maestro criteria have a predictive value in the preoperative planning for this percutaneous technique?. *BioMed Research International*.

[10] Hanyu T., Yamazaki H., Murasawa A., Tohyama C. (1997). Arthroplasty for rheumatoid forefoot deformities by a shortening oblique osteotomy. *Clinical Orthopaedics and Related Research*.

[11] Niki H., Hirano T., Okada H., Beppu M. (2010). Combination joint-preserving surgery for forefoot deformity in patients with rheumatoid arthritis. *Journal of Bone and Joint Surgery. British Volume*.

[12] Choi J. Y., Lee J. M., Suh J. S. (2019). Shortening proximal chevron metatarsal osteotomy for patients with a hallux valgus deformity with advanced arthritis. *The Journal of Foot and Ankle Surgery*.

